# Risk Factors for Orbital Invasion in Malignant Eyelid Tumors, Is Orbital Exenteration Still Necessary?

**DOI:** 10.3390/jcm13030726

**Published:** 2024-01-26

**Authors:** Krzysztof Gąsiorowski, Michał Gontarz, Tomasz Marecik, Paweł Szczurowski, Jakub Bargiel, Jan Zapała, Grażyna Wyszyńska-Pawelec

**Affiliations:** Department of Cranio-Maxillofacial Surgery, Jagiellonian University Medical College, 30-688 Cracow, Poland; michal.gontarz@uj.edu.pl (M.G.); tomasz.marecik@uj.edu.pl (T.M.); pawel.szczurowski@uj.edu.pl (P.S.); jakub.bargiel@uj.edu.pl (J.B.); jan.zapala@uj.edu.pl (J.Z.); grazyna.wyszynska-pawelec@uj.edu.pl (G.W.-P.)

**Keywords:** orbital exenteration, eyelid tumors, periocular BCC, periocular malignancy

## Abstract

Basal cell carcinoma is the most common malignant skin tumor of the eyelids in Caucasians, followed by squamous cell carcinoma and sebaceous gland carcinoma. The primary treatment for these tumors is radical excision. In cases where malignant eyelid tumors are advanced and have invaded the orbit, orbital exenteration is necessary. In this retrospective study, we aimed to determine the correlation between the risk of orbital infiltration and various factors like tumor location, size, histological type, and patient age. This study revealed that tumors in multiple regions increased the risk of orbital infiltration by 3.75 times. Tumors with a diameter of 21–30 mm raised the likelihood of requiring exenteration by 15.5 times compared to smaller tumors (up to 10 mm). Age was also associated with the likelihood of orbital invasion in periocular tumors. Interestingly, no correlation was found between the histological type of the tumor and the risk of orbital infiltration. Notably, the conjunctiva of the eyeball was the most commonly infiltrated orbital structure, followed by the orbital fat. Timely treatment and well-planned procedures are crucial for patients with malignant periocular skin tumors to avoid multiple reoperations and the potential need for orbital exenteration.

## 1. Introduction

The most common malignant skin tumor of the eyelids in the Caucasian race is basal cell carcinoma (BCC), comprising nearly 85% of malignant eyelid tumors. Less common is squamous cell carcinoma (SCC), comprising 10%, and the rarest are sebaceous gland carcinoma (SGC) and malignant melanoma [1,2].

The etiopathogenesis of malignant eyelid tumors varies and depends on the type of tumor. Intermittent and intense exposure to ultraviolet (UV) radiation is a well-recognized and crucial risk factor for the development of basal cell carcinoma (BCC). Specifically, shorter-wavelength UVB radiation, which falls within the range of 290–320 nm and is often associated with sunburn, plays a more prominent role in the formation of BCC compared to longer-wavelength UVA radiation, ranging from 320 to 400 nm, and often associated with tanning. [1,2,3]

Immunosuppression is a recognized and established risk factor for the development of squamous cell carcinoma (SCC). Cases of SCC occurring in the eyelid have been documented in individuals who have undergone renal transplantation and in those with human immunodeficiency virus (HIV) infection [4].

Most periocular sebaceous gland carcinomas (SGC) typically originate from the meibomian glands situated within the tarsus. Periocular sebaceous gland carcinoma (SGC) predominantly originates in the upper eyelid, representing approximately 50% to 66% of all cases. This higher incidence in the upper eyelid is attributed to the greater concentration of meibomian glands found in this area [5,6].

The main purpose of treatment is the radical excision of the tumor to achieve to the best local control. If treatment is delayed, periocular malignancies can infiltrate the orbit and the skull base or spread to regional lymph nodes [7]. Advanced malignant eyelid tumors with orbital invasion indicate the need for orbital exenteration and in most cases require postoperative radiotherapy [7,8].

However, the improved likelihood of survival after orbital exenteration is debated, as most of the studies dealing with this problem are retrospective. [9] The risk factors for orbital invasion include elderly male patients, advanced stage of the tumors, local recurrences, localization in the medial canthal area, aggressive histological subtypes, and perineural invasion [3,9].

The aim of this study is an assessment between the risk of orbital infiltration and the location, stage, and histological type of periorbital malignant eyelid skin tumors in the clinical data of patients of the study.

## 2. Materials and Methods

Retrospective analysis of a group of 179 patients with periocular nonmelanotic skin cancers operated on in the Department of Cranio-Maxillofacial Surgery of the Jagiellonian University in Cracow between January 2003 and December 2020 was performed. Among this group, exenteration was performed in 42 patients. Only patients with primary eyelid nonmelanotic skin cancers were included in the study group. The database comprised the following: age, sex, location of the lesion, advancement, CT or MRI imaging, the result of the histopathological examination with particular emphasis on the infiltration of the specific orbital content, postoperative complications, and the follow up. Excised lesions were verified according to the 2017 WHO classification. Based on general health condition, location of the lesion, and extent of the procedure, the decision on immediate or delayed reconstruction was made.

The analysis of qualitative variables was performed by calculating the number and percentage of occurrences of each value. The comparison of the values of the qualitative variables in the groups was performed using a chi-square test (with Yates’s correction for 2 × 2 tables) or a Fisher’s exact test, where low expected frequencies appeared. A significance level of 0.05 was adopted in the analysis. The analysis was performed in an R program, version 4.2.2.

## 3. Results

In this study, there were 90 males (50.3%) and 89 females (49.7%). The average age of the patients was 69 years. Basal cell carcinoma (BCC) was the most prevalent skin cancer in the periocular region, with 170 patients, while squamous cell carcinoma (SCC) was less common, with 8 patients, and there was only one case of sebaceous gland carcinoma (SGC).

Patients included in this study during the first phase were managed in an outpatient clinic where biopsy samples for histopathological examination were taken from skin ulcers in the periorbital region. Additionally, in cases where orbital bone infiltration was suspected, imaging studies such as Computed Tomography (CT) or Magnetic Resonance Imaging (MRI) were made. Decisions regarding the method of reconstruction were made depending on the patient’s overall health status, the extent of the lesion, and the feasibility of postoperative defect reconstruction. The entire diagnostic process prior to admission to the clinic did not exceed one month.

Among the patients diagnosed with basal cell carcinoma (BCC), there were 64 cases of nonaggressive subtypes, 23 cases of aggressive subtypes, and the subtype of BCC was not specified by the pathologist in 83 cases. The histopathological subtypes of squamous cell carcinoma (SCC) were not analyzed in this study. In terms of surgical outcomes, complete (R0) resection was achieved in 80 patients, nonradical excision in 47 patients, and in 52 patients, the histopathologist noted excisions with a margin of less than 3 mm. Among the group of patients with significant local advancement who underwent exenteration, radical treatment was achieved in 26 patients, excision with a narrow margin in 5 patients, and R1 resection was performed in 11 patients.

Table 1 shows the study group characteristics according to age, sex, localization, histopathological subtype, and recurrences.

The most frequent tumor localization was observed in the medial canthus, affecting 84 patients, followed by the lower eyelid in 62 patients.. However, due to the substantial advancement of neoplasms, the precise determination of the primary tumor focus location was not possible in 14 patients.

**Figure 1:**
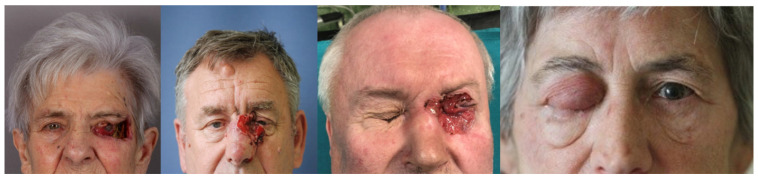
Local advancement of the tumors in patients requiring orbital exenteration. The rightmost image shows sebaceous gland carcinoma of the upper eyelid resembling eyelid inflammation.

In the group of patients requiring exenteration, the most prevalent symptom was ulceration adhering to the orbital bone (Figure 1).

The second most common symptom was ocular motility disturbance, which occurred in 13 patients (30.9%), followed by epiphora resulting from lacrimal duct infiltration. There was only one case where the tumor resembled inflammation (Figure 1), which was sebaceous gland carcinoma (SGC). Postexenteration recurrence was identified in three patients, with a mean time of 16 months (ranging from 6 to 23 months). The recurrence rate after orbital exenteration was 7.14%. All patients with recurrence underwent extensive surgical procedures followed by postoperative radiotherapy.

Imaging studies, including computed tomography (CT) and magnetic resonance imaging (MRI), were conducted on patients in whom clinical examination and ophthalmologic assessment raised suspicions of orbital invasion. Among the patients who underwent exenteration, CT scans were carried out in 36 cases, while MRI scans were performed in 2 casesThere were no discrepancies noted between the reported orbital invasion in the imaging studies and the specific structures infiltrated, as described in the histopathological examination. The limited number of imaging studies conducted on patients with malignant eyelid skin tumors can be attributed to the retrospective nature of this study and the limited availability of imaging modalities, particularly MRI, in the early years of the 21st century. In cases where local recurrence was suspected, imaging studies were performed to assess the potential for orbital invasion and to plan the appropriate extent of tumor resection.

According to this study, the likelihood of orbital infiltration was significantly higher (*p* < 0.05) in patients with tumors originating in the lower eyelid compared to other locations. Additionally, neoplasms arising in more than one region increased the risk of orbital infiltration by 3.75 times. Table 2 illustrates the correlation between the lesion’s location and orbital infiltration.

Another significant factor that considerably increased the risk of orbital infiltration and, consequently, the need for exenteration, was the diameter of the tumor. In our study, we observed that the risk of orbital infiltration in tumors with a diameter of 21–30 mm increased the likelihood of exenteration by 15.5 times compared to tumors with a diameter of up to 10 mm. Furthermore, tumors with a diameter exceeding 30 mm increased this risk by 71 times. We also found a correlation between the patient’s age and the likelihood of orbital invasion in periocular tumors. Among patients in their seventh decade of life, the risk of orbital infiltration was 3.84 times higher, and among patients in their eighth decade of life, the risk of orbital infiltration was 5.59 times higher than in patients under 60 years of age (Table 2).

With the advancement of the lesion, the risk of nonradical excision increases, which is particularly common in cases of malignant tumors located in the area of the medial canthal angle. This is especially noticeable in basal cell carcinomas, where nonradical excision statistically more often leads to recurrence compared to narrow-margin excision of BCC. Moreover, the histological subtype of the tumor also influences the risk of nonradical excision [10].

No significant correlation was observed between the histological type of the tumor and the risk of orbital infiltration. However, aggressive subtypes of basal cell carcinoma (BCC) and squamous cell carcinoma (SCC) statistically increase the risk of nonradical excision (*p* = 0.003) of periorbital neoplasms, which could lead to recurrence with infiltration of orbital content.

There was no correlation found between the location of the primary lesion and the likelihood of nonradical excision of the tumor.

The most commonly infiltrated orbital structure was the conjunctiva of the eyeball, followed by orbital fat (21 cases), orbital periosteum, extraocular muscles, and the eyeball. Infiltration of the lacrimal gland was found in only two cases. No statistically significant correlation was identified between the tumor’s location and the infiltrated orbital structures.

## 4. Discussion

Basal cell carcinoma (BCC) of the eyelid is the most prevalent periocular skin neoplasm among the Caucasian population, constituting 80–90% of periocular malignancies. Less common are squamous cell carcinoma (SCC) at 5–10%, followed by sebaceous gland carcinoma (SGC) and cutaneous melanoma [1]. Despite its slow development, BCC in the periocular region accounts for almost 50% of exenterations, as reported by Tyers [7]. Other malignancies, such as SCC and SGC, exhibit infiltrative growth patterns and carry a significantly higher risk of lymph node involvement, accounting for 1–25% and 10–25%, respectively, for SGC [1,5]. 

UVB radiation is particularly harmful as it causes damage to DNA and impairs the body’s repair mechanisms. A significant finding is that approximately 50% of BCC cases exhibit mutations in the TP53 tumor-suppressor gene induced by UV exposure. These mutations play a crucial role in the process of skin carcinogenesis, activating genes within the hedgehog intercellular signaling pathway, such as patched (Ptch), sonic hedgehog, and smoothened. In particular, Ptch-1 mutations have been associated with the development of eyelid BCC. Furthermore, prolonged exposure to UV radiation can induce Ptch-1 mutations over time, further promoting the development of BCC [11].

Squamous cell carcinoma (SCC), notably the second most common type of skin cancer affecting the eyelid and surrounding eye area in Western countries, ranks as the third most frequent malignant eyelid tumor in India and China, as evidenced by extensive epidemiological studies [1,2,7]. SCC is distinguished by its rapid growth rate, extensive subclinical spread beneath the skin’s surface, and a high likelihood of invading nearby nerves and blood vessels [1]. Critical complications associated with periocular SCC encompass the spread of the cancer into the eye socket and brain, the potential for cancer cells to spread to lymph nodes and distant parts of the body, and an increased risk of death. [12] Immunosuppression plays a significant role in the development of SCC and SGC. Moreover, the risk of cutaneous SCC can be influenced by the emergence of immune suppression, which occurs in patients with HIV, hematological neoplasms, or autoimmune diseases treated with immunosuppression [4].

Sebaceous gland carcinoma is an adnexal tumor that usually occurs in the periocular region, mostly in elderly females. SGC arises from the Meibomian glands of the tarsus and the Zeis glands which occur more often in the upper eyelid. It can rarely arise from the caruncle (5%). The nodular and spreading types of SGC are the two main pathological varieties [13]. The nodular type usually presents as a well circumscribed nodule with adipose deposits in the upper tarsal plate. The spreading type of SGC involves the eyelid margin, causing loss of eyelashes, and can easily be misdiagnosed as blepharoconjunctivitis. The clinical diagnosis of SGC can be difficult because it mimics other benign eyelid lesions, such as chronic blepharitis, chronic blepharoconjunctivitis, and recurrent chalazion [14].

In our dataset, the majority of patients had periocular basal cell carcinoma (BCC), accounting for 95% of cases, while periocular squamous cell carcinoma (SCC) was present in 4.5% of patients. We observed only one case of periocular sebaceous gland carcinoma (SGC), which represented 0.5% of cases. Our findings align with the results reported by other authors in the literature [2,15]. It is worth noting that, in contrast, SGC is reported as the most common or second most common periocular malignancy in the Asian population, according to Slutsky et al. [1,2]. 

The incidence of periocular basal cell carcinoma (BCC), squamous cell carcinoma (SCC), and sebaceous gland carcinoma (SGC) tends to increase with age, with the highest occurrence observed between the sixth and eighth decades of life. These malignancies are slightly more frequent in men [6,16], which is consistent with the findings of this study where the average patient age was 69 years. However, in this study, the male-to-female ratio was 1:1. 

Nonmelanoma eyelid skin malignancies are associated with a 2–4% risk of orbital invasion [3,7,17]. The likelihood of orbital infiltration increases in cases of multiple recurrences, malignant tumor subtypes, and when the primary focus is located in the lower eyelid or medial angle of the eye [8]. In this study, the percentage of patients with orbital infiltration requiring exenteration was notably higher, accounting for 23.4%, compared to the study by Furdova et al. [17]. 

The high incidence of orbital infiltration in the presented study is likely attributed to the fact that the vast majority of patients in the study group met the criteria associated with a higher risk of orbital infiltration. These risk factors include aggressive basal cell carcinoma (BCC) subtypes, older age (with a mean age of 74 years), and the primary tumor focus being located in the lower eyelid and medial canthus. Additionally, the average duration from the onset of initial symptoms to obtaining a histopathological sample was 80 months, with a range spanning from 12 to 240 months. 

According to the literature, aggressive subtypes of basal cell carcinoma (BCC), such as infiltrative, morpheaform, and basosquamous, are more likely to lead to orbital invasion [18]. However, in this study, no correlation was observed between aggressive subtypes of BCC and a higher risk of orbital infiltration (*p* = 0.38). Nevertheless, this study did confirm a correlation between aggressive subtypes and the risk of nonradical excision, which is one of the risk factors for orbital infiltration (*p* = 0.003). 

The most common symptom of periocular malignancies in this study was a slowly growing nodule that eventually progressed into an ulcer, which aligns with findings from other studies [1,2]. Clinical signs of orbital invasion typically involve the attachment of a mass to the orbital rim, reduced eye motility, displacement of the eyeball, and, in cases where the medial canthus is affected, excessive tearing due to involvement of the lacrimal drainage system [3]. 

In advanced stages of periocular basal cell carcinoma (BCC), squamous cell carcinoma (SCC), and sebaceous gland carcinoma (SGC), the most common treatment approach involves surgery with intraoperative frozen-section margin control. Mohs micrographic surgery (MMS) is generally not suitable for cases involving extensive orbital invasion, as it presents challenges in obtaining well-oriented and flat specimens from soft tissues and periosteum within the orbit [3]. 

When it is not possible to achieve clear margins during the excision, or when the excision margins are in close proximity to the tumor, there is a compelling reason to consider adjuvant radiotherapy as a complementary treatment. The recurrence rate after exenteration in this study was 7.8%, which is consistent with findings from other studies [3,18,19,20]. 

In cases of locally advanced, unresectable tumors infiltrating the skull base, the use of Vismodegib, a selective hedgehog pathway inhibitor, may be considered as one of the treatment methods [21,22]. 

Due to fact that mutations in the hedgehog pathway’s genes have been associated with the development of BCC, hedgehog pathway inhibitors (hHi) have proven to be effective in treating patients with locally advanced BCC. Vismodegib and sonidegib are oral medications that inhibit the hedgehog pathway by targeting the transmembrane protein Smoothened (SMO). They have received different approvals from the US Food and Drug Administration (FDA) and the European Medicines Agency (EMA) [23]. Both vismodegib and sonidegib function by inhibiting the hedgehog (HH) pathway through the antagonism of SMO, resulting in comparable safety profiles. They have demonstrated effectiveness in locally advanced BCCs (laBCCs), but common side effects, such as muscle spasms, dysgeusia, alopecia, weight loss, and fatigue, are frequently observed [24]. Recently, Patidegib, a novel hedgehog inhibitor (hHi), was evaluated in a topical gel formulation at 4% and 2% concentrations, successfully completing its phase three clinical trial with promising results. Patidegib shares the same mechanism as Vismodegib and Sonidegib, but its topical application presents a potentially more favorable option, especially for elderly patients and those who have encountered severe side effects from other systemic hHi treatments [25].

According to Ching et al., due to the fact that hedgehog pathway inhibitors (HHIs) primarily suppress rather than cure basal cell carcinoma (BCC), it is recommended for use in conjunction with definitive surgical resection. This combined approach allows for optimal reduction in tumor invasion, enabling a less invasive surgical resection and subsequent reconstruction, which is often associated with fewer complications and improved outcomes [26].

Immune checkpoint inhibitors offer a novel treatment approach for cutaneous squamous cell carcinoma (cSCC) that is surgically unresectable and presents with locally advanced or distant metastatic disease. Cemiplimab, in particular, received approval from the US Food and Drug Administration (FDA) and the European Commission and advisory committees of the National Institute for Health and Care Excellence for the treatment of locally advanced or metastatic cSCC cases that do not meet the criteria for curative surgical resection or curative radiotherapy [12] In a pivotal study led by Migden et al. focusing on immunotherapy in metastatic cSCC, cemiplimab, which is a monoclonal antibody targeting the PD-1 receptor pathway, demonstrated an impressive 50% response rate [27]. Additionally, there is emerging but limited evidence suggesting that neoadjuvant PD-1 inhibitor immunotherapy might be a potential option for locoregionally advanced cSCC that is amenable to surgical resection [28].

In the reviewed literature, globe-sparing surgical excision is described, but it is typically reserved for specific cases, such as patients with poor vision in the opposite eye, patients with a single remaining eye, and cases with anterior orbital involvement [3]. However, this approach necessitates regular MRI examinations, which should be conducted at least annually for a minimum of 5 years following the initial postoperative evaluation [3,29].

## 5. Conclusions

The treatment of locally advanced malignant eyelid tumors is challenging. Despite the typically slow growth of basal cell carcinoma (BCC) and the relatively rare occurrence of squamous cell carcinoma (SCC) and sebaceous gland carcinoma (SGC) in the periocular region, patients with malignant periocular skin tumors require specialized care. Delayed treatment initiation and inadequately planned procedures leading to multiple reoperations can result in the need for orbital exenteration and, in some instances, an increased risk of mortality due to inoperable recurrences infiltrating the skull base. It is crucial to remain vigilant, as orbital invasion can develop without obvious symptoms. Therefore, careful consideration of the extent of resection based on MRI or CT imaging is essential when planning surgeries for malignant eyelid skin tumors. It is worth noting that immunotherapy with Cemiplimab and the use of hedgehog pathway blockers, whether administered systemically (Vismodegib, Sonidegib) or topically (Patidegib), present potential treatment options. However, it is essential to emphasize that the current knowledge is limited, and there is a lack of published research in this specific area, as well as a shortage of prospective studies. Therefore, further investigations are warranted to evaluate the efficacy and safety of these therapeutic modalities.

## Figures and Tables

**Table 1:** Study group characteristics according to orbital infiltration.

Parameter	Non orbital Infiltration	Orbital Infiltration	*p*-Value
Mean age:	67 (20–95)	74 (51–93)	*p* > 0.05
Sex M:F	72:65	18:24	*p* > 0.05
Localization: - Medial canthal region - Lower Eyelid - Multiple locations - Upper Eyelid - Lanteral canthal region	70 (51.1%) 42 (30.6%) 8 (5.8%) 10 (7.4%) 7 (5.1%)	14 (33.3%) 20 (47.6%) 6 (14.5%) 1 (2.3%) 1 (2.3%)	0.03 0.031
Histopathological type: - Basal cell carcinoma - Squamous cell carcinoma	137 (76.5%) 0	34 (19%) 8 (4.5%)	*p* > 0.05
Recurrences	4 (2.9%)	3 (7.1%)	*p* > 0.05

**Table 2:** Analysis of risk factors for orbital exenteration.

Localization	Infiltration of the Orbital Content (Exenteration)	OR	95% CI		*p*
Medial canthus (No = 84)	14 (16.67%)	1	ref.		
Lower eyelid (No = 62)	20 (32.26%)	2.381	1.088		5.209
Upper eyelid (No = 10)	1 (10.00%)	0.556	0.065		4.741
Lateral canthus(No = 9)	1 (11.11%)	0.625	0.072		5.401
Unknown starting point, multiple locations (No = 14)	6 (42.86%)	3.75	1.125		12.501
Localization	Infiltration of the orbital content (exenteration)	OR	95% CI		*p*
Medial canthus (No = 84)	14 (16.67%)	1	ref.		
Lower eyelid (No = 62)	20 (32.26%)	2.381	1.088	5.209	0.03
Upper eyelid (No = 10)	1 (10.00%)	0.556	0.065	4.741	0.591
Lateral canthus(No = 9)	1 (11.11%)	0.625	0.072	5.401	0.669
Unknown starting point, multiple locations (No = 14)	6 (42.86%)	3.75	1.125	12.501	0.031
Diameter of tumor	Infiltration of orbital content (exenteration)	OR	95% CI		*p*
≤10 mm (N = 64)	2 (3.12%)	1	ref.		
11–20 mm (N = 54)	7 (12.96%)	4.617	0.917	23.25	0.064
21–30 mm (N = 27)	9 (33.33%)	15.5	3.069	78.287	0.001
>30 mm (N = 33)	23 (69.70%)	71.3	14.514	350.27	<0.001
Patients’ age at the time of the surgery	Orbital infiltration	OR	95% CI		*p*
60 y.o (N = 45)	4 (8.89%)	1	ref.		
61–70 y.o (N = 42)	8 (19.05%)	2.412	0.668	8.702	0.179
71–80 y.o (N = 55)	15 (27.27%)	3.844	1.174	12.579	0.026
>80 y.o (N = 34)	12 (35.29%)	5.591	1.611	19.403	0.007

## Data Availability

Restrictions apply to the availability of these data. Data were obtained from patients treated at the Department of Cranio-Maxillofacial Surgery, Cracow, Poland, and cannot be shared, in accordance with the General Data Protection Regulation (EU) 2016/679.
